# Brightening Medical Reports

**Published:** 1921-05-07

**Authors:** 


### THE PUBLIC WELL-BEING.

### Brightening Medical Reports.

A medical correspondent, favourably impressed with the account given in this column of methods by which questions of public health can be made easily available, as well as intelligible and interesting to the whole community, writes to say that he should like to add one more proposal to the list, and that is that the medical officers of health throughout the country should be requested to present their reports of local conditions in a much more attractive form than is commonly adopted. As a rule, he asserts, these reports are as dull as the dullest sermons, to which nobody pays any attention, outside a small body of experts whose business it is to read them. As an example of how such reports should be framed, he sends us a number of racy extracts from the annual report of Dr. J. B. Yeoman, medical officer of health for Wirral, who comments with pungent force upon the insanitary conditions of the bungalow town at Moreton, near Birkenhead. This enterprising official opens with the observation that the poet's haunting line "O! fair is Moreton-in-the-Marsh," does not apply to Moreton, near Birkenhead. Even Moses, he adds, "the first medical officer of health," would not have permitted the methods adopted at Moreton for disposing of refuse within his camp. Had it been the lot of Moses to work in the Moreton fields instead of the wilderness his regulations would have penalised any burial of excreta within a mile of the encampment, in support of which he quotes Deut. xxii. 12: "Thou shalt have a place also without the camp, whither thou shalt go forth abroad."

After such an opening everybody at Moreton, near Birkenhead, must surely have been compelled to read the whole story of the indictment. Dr. Yeoman, it is evident, quite consciously tries to be picturesque in order to drive home his health lessons. For, commenting in the same document on annual reports in general, he states that they are supposed to be of an educational character, but there is no compulsion upon persons to read them, with the result that they are very rarely, if ever, perused by the individuals whom they most concern. "It is conceivable," he continues, "that some day a medical officer will write his report in terms of the betting-ring, so as to reach the sporting section of the community. For example, he might state that it is 100 to 1 that the man who is constantly nipping will get cirrhosis of his liver, or that it is 50 to 1 that the man who exhausts himself and lives in unventilated rooms on an unsatisfactory diet will acquire tuberculosis, or that it is a 'dead cert.' that the three-months'-old infant fed upon boiled bread will suffer from rickets later on."

"There can be no question," the doctor adds, "that the national health conscience is slowly being evolved, but it is lethargic, sluggish, and tardy in its action, and requires to be stimulated, directed, and trained in order that liveliness may be produced. In epidemic times, such as influenza years and infantile diarrhœa seasons, or in war years, it is active, but in years like 1920 there is a tendency to 'fold our hands and suck our gums, and think well of it,' as in the following criticism on the Registrar-General's figures: 'These figures are so encouraging that we may reasonably hope they will put an end to the silly talk about a dying and degenerate race. The race is healthy and sound; it stands, on this showing, second to none on earth.' Yet the writer of those lines knew that in 1919 there were 46,312 deaths attributable to tuberculosis, 44,801 deaths due to influenza, 42,144 deaths from cancer, 38,949 deaths from pneumonia, and 51,530 deaths from organic heart disease. He knew that 98,000 new patients attended at venereal disease clinics. He was aware that sickness and disablement benefit cost the country in actual cash over £5,500,000 per annum, and a very large amount of the sickness represented by these payments could be prevented."

This is sound sense, admirably expressed. We congratulate Wirral on one of the best annual medical reports we have ever read. May Dr. Yeoman continue his good work, and may some of his less enterprising and less unconventional colleagues be inspired to imitate him.

